# Efficacy of the Triple-Dose Prophylactic Vitamin K Regimen in Healthy Neonates and Evaluation of the Utility of Vitamin K Deficiency Screening

**DOI:** 10.7759/cureus.74436

**Published:** 2024-11-25

**Authors:** Ryo Matsuoka, Emi Nonaka, Satoshi Fujita, Naoe Akiyama

**Affiliations:** 1 Department of Pediatrics, Fuji City General Hospital, Fuji, JPN; 2 Department of Pediatrics, Jikei University School of Medicine, Tokyo, JPN

**Keywords:** hepaplastin test, menaquinone-4, newborn screening, triple-dose regimen, vitamin k2, vitamin k deficiency bleeding, vitamin k prophylaxis

## Abstract

Background: In Japan, three doses of vitamin K are administered to neonates as prophylactic regimens against vitamin K deficiency bleeding (VKDB). In this study, we aimed to evaluate the efficacy of this prophylactic vitamin K regimen using the hepaplastin test (HPT) performed one, two weeks, and one month after birth. The secondary aim of this study was to evaluate the utility of HPT screening in healthy neonates.

Method: This study included a retrospective analysis of HPT values in neonates born between June 2009 and February 2018 using the prophylactic regimen implemented in 2011.

Results: The study group included 6075 neonates, of whom 274 (4.5%) had a low HPT value (<40%) at the time of discharge (approximately one week after birth). Follow-up HPT was performed in 118 neonates at two weeks, with a low HPT value persisting in 11 neonates (9%). There was no effect of breast or formula milk on HPT values, and all neonates achieved an HPT value >40% at one month, regardless of whether vitamin K supplementation was provided at two weeks. None of the infants had underlying diseases that led to secondary VKDB.

Conclusion: Healthy newborns maintained adequate HPT values with the triple-dose vitamin K administration, regardless of the feeding method. Therefore, HPT screening might not be essential for healthy neonates.

## Introduction

Vitamin K plays a crucial role in the synthesis and activation of coagulation factors II (prothrombin), VII, IX, and X, which are vitamin K-dependent, along with proteins C and S [1]. There are three recognized forms of vitamin K: vitamin K1 (phylloquinone), vitamin K2 (menaquinone), and vitamin K3 (menadione) [2]. Inadequate placental transfer, low vitamin K content in breast milk, and immature gut flora impair intestinal absorption, rendering infants susceptible to vitamin K deficiency [3,4]. Vitamin K deficiency can lead to severe hemorrhagic diseases (Vitamin K deficiency bleeding; VKDB), such as intracranial hemorrhage, which can pose life-threatening risks. Hence, prophylactic vitamin K should be administered to all newborns and infants, although the specific method of administration may vary among different countries [5].

 In 2010 and 2011, guidelines [6] and modified guidelines [7] for VKDB in infants and newborns were published by the Pediatric Society of Japan, which recommended the administration of oral vitamin K to full-term neonates. In Japan, vitamin K2 (menaquinone-4) syrup is typically administered as a preventive measure. The modified guidelines recommend that the vitamin K regimen include one dose at birth, one before discharge from the obstetrics department (approximately one week of age), and a third dose on the one-month medical checkup [7]. Since then, this triple-dose regimen of vitamin K prophylaxis has become standard practice in Japan. However, despite this vitamin K regimen, VKDB continues to be reported [8]. As VKBD can develop secondary to hepatobiliary diseases such as biliary atresia and impaired gastrointestinal absorption of vitamin K, as well as idiopathic vitamin K deficiency, the effectiveness of the oral triple-dose vitamin K regimen remains to be fully clarified.

Therefore, this study aimed to evaluate the efficacy of a triple-dose vitamin K regimen by retrospectively analyzing the outcomes of vitamin K screening using the hepaplastin test (HPT). To date, the preventive effect of vitamin K deficiency from the reported triple-dose regimen has been assessed based on the incidence of VKDB [5]. In this study, we expected to obtain specific results by evaluating HPT screening, which would enable us to assess latent vitamin K deficiency. HPT screening for VKDB has routinely been performed in Shizuoka Prefecture since its introduction in 1986, according to the guidelines of the Shizuoka Prefectural Policy Committee for the Prevention of VKDB [9-11]. By performing HPT screening, it was hoped that the risk of vitamin K deficiency could be identified in advance, allowing for more opportunities to explore organic diseases thoroughly and consider additional vitamin K administration. The secondary aim of this study was to evaluate the effectiveness of HPT screening.

## Materials and methods

Description of the study group

The medical records of all neonates born at Fuji City General Hospital between June 2009 and February 2018 were retrospectively reviewed to exclude all neonates admitted to the neonatal intensive care unit, born prematurely, and/or with low birth weight, as well as those who received vitamin K injections (rather than vitamin K syrup). Also, patients who received antibiotic treatment were excluded, as it affects intestinal bacteria and subsequently influences vitamin K metabolism [12,13]. 

Blood samples for HPT were obtained from the heel and were typically collected before feeding. The HPT was performed using blood samples obtained from the heel and measured using a coagulation (factors II, VII, and X) deficiency plasma kit (Compound Factor H Assay; Sysmex Corporation, Kobe, Japan), with correction for the hematocrit value. According to the guidelines of the Shizuoka Prefectural Policy Committee for the prevention of VKDB [9], vitamin K deficiency was defined as an HPT value of <40%, with therapeutic thresholds set at <29% for 1-week screening and <37% for 1-month screening [9-11, 14, 15]. The feeding method (breast milk or formula milk/mixed feeding) at the time of HPT screening was extracted from the interview sheet completed during the 1-month medical checkup. 

The vitamin K prophylaxis protocol at Fuji City General Hospital

**Figure 1:**
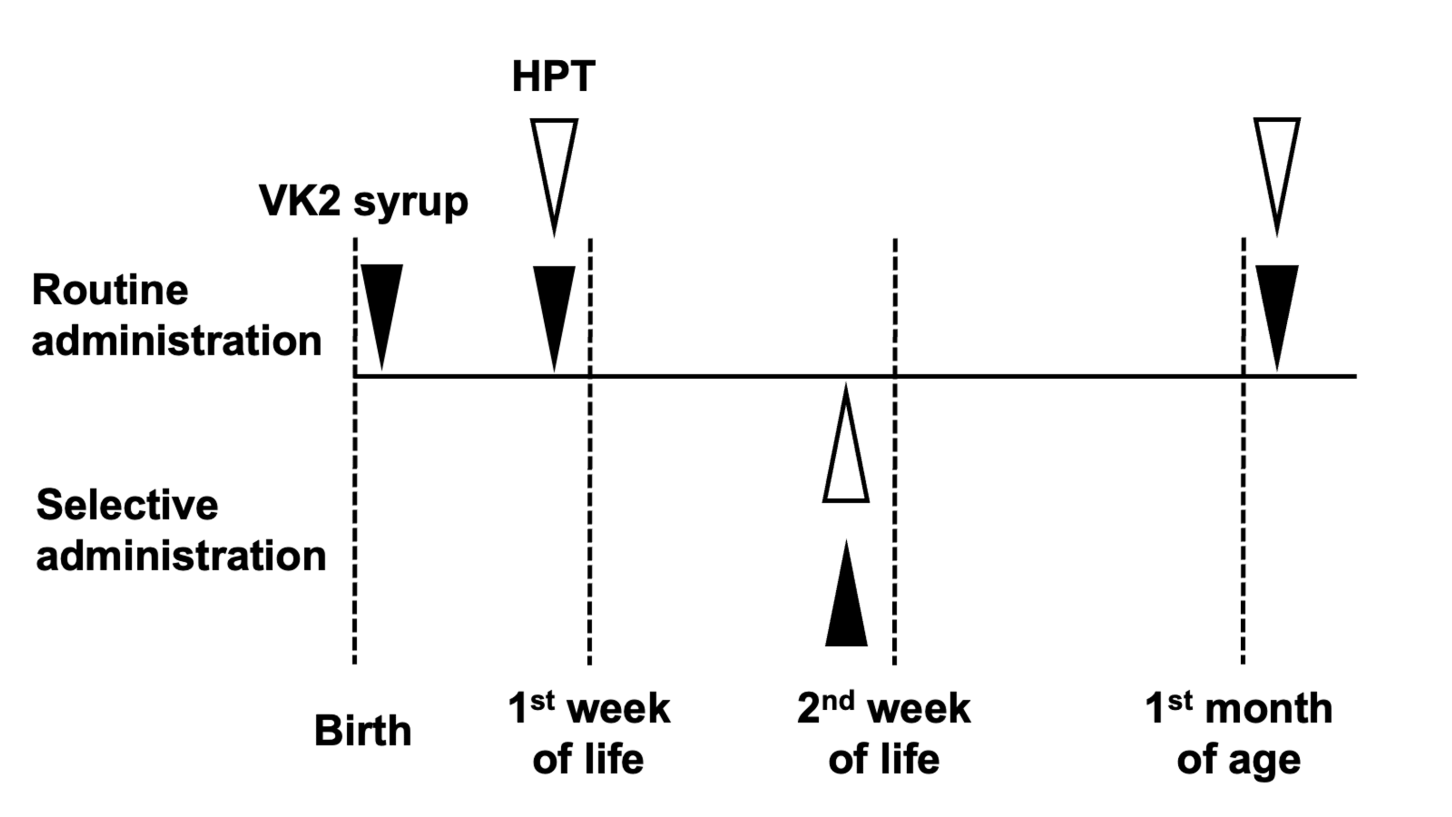
Vitamin K prophylaxis protocol at Fuji City General Hospital. Routine administration of three doses of vitamin K2 syrup (black arrows) in combination with HPT screening (white arrows) at post-natal week one (through our pediatric outpatient clinic). Follow-up screening at the one-month health checkup is at the attending physician's discretion. Neonates with an HPT of <40% at the one-week screen are referred for further follow-up at post-natal week two.

Metetrenone-4 vitamin K2 syrup (KaytwoTM, Eisai Corporation Ltd, Tokyo) was administered orally to all neonates three times: after birth; once feeding had been established, before discharge from the obstetrics department (approximately one week after birth); and at the one-month medical checkup. All doses were administered at the hospital, and no failures were noted. HPT screening was routinely performed at discharge and during the 1-month checkup before vitamin K administration. Infants with a low HPT (<40%) at discharge were referred for a follow-up at the pediatric outpatient department. Reexamination of the HPT, additional oral vitamin K, and a follow-up until the one-month medical examination were left to the attending physician’s discretion (Figure 1).

Statistical analysis

Data are presented as the means±SEM and were analyzed using GraphPad Prism ver. 9.5.0 (GraphPad Software Inc., San Diego, Calif, USA). Two-group datasets were analyzed using one-way ANOVA variance with the Mann-Whitney U test. A p-value ≤0.05 was considered statistically significant.

Ethical considerations

All procedures followed the ethical standards of the responsible committee on human experimentation (institutional and national), the Helsinki Declaration of 1964, and later versions. The ethical aspects of this study were reviewed and approved by the Institutional Review Board of Fuji City General Hospital in 2018 (#179).

## Results

**Figure 2:**
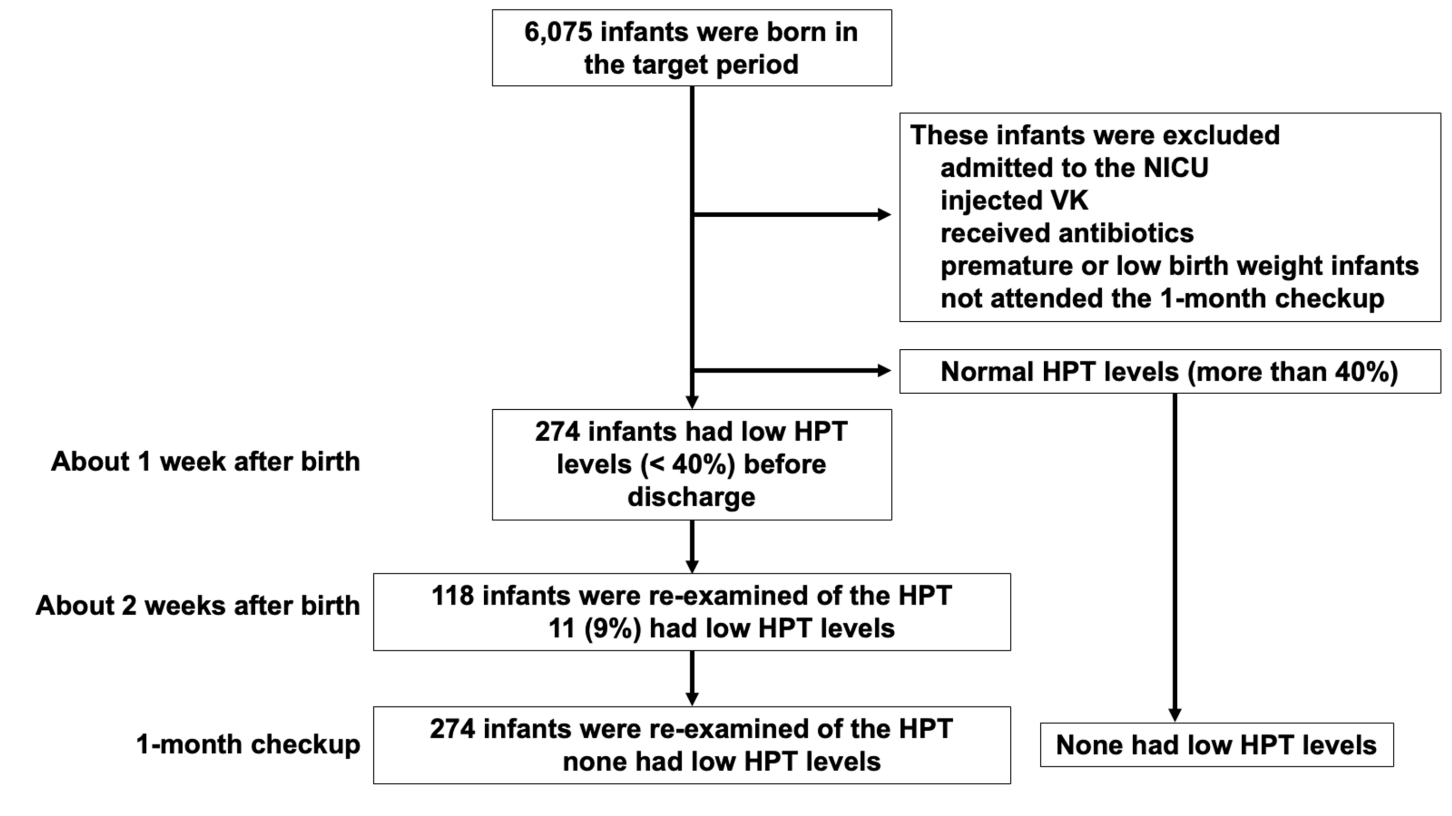
Assessment of study participants and outcomes. Neonatal intensive care unit, NICU.

 Over the study period, all 6075 births were registered at the Fuji City General Hospital. Among them, 274 (4.5%) had an HPT value <40% at discharge (approximately one week after birth). In this group, HPT was reevaluated in 118 neonates two weeks after birth, with a low HPT value (<40%) persisting in 11 neonates (9%). Notably, all infants with persistently low HPT values at two weeks were above the therapeutic threshold of 30%. At the one-month screening, the HPT values were normal (>40%) in all infants (Figure 2). 

**Figure 3:**
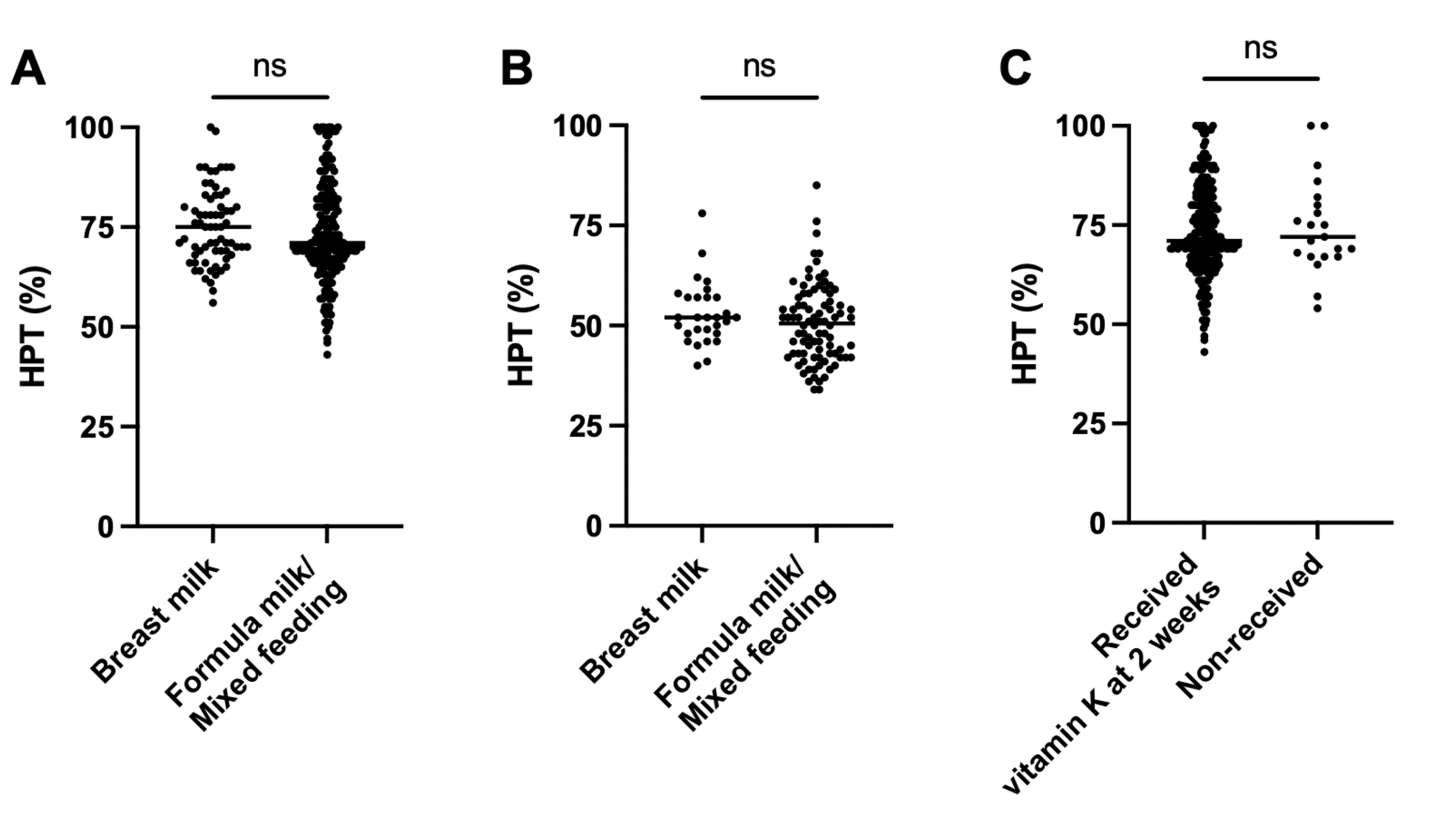
HPT values for each period after birth. (A) Differences in HPT values at one month for the breast and formula milk/mixed feeding groups. (B) Differences in HPT values at two weeks for the breast and formula milk/mixed feeding groups. (C) Differences in HPT values at one month between neonates who received vitamin K supplementation at week two and those who did not.

The HPT values at two weeks and one month after birth were plotted against the feeding method (breast milk or formula milk/mixed feeding) in Figure 3A and Figure 3B, respectively, with no differences between the two groups (p=0.230 and p=0.188). Figure 3C compares the HPT values at one month among neonates who received additional vitamin K at two weeks and those who were followed without additional vitamin K administration. At the two-week check-up for neonates in the low HPT group, the decision to administer vitamin K was left to the discretion of the attending physician, with 253 (93.3%) neonates ultimately receiving vitamin K. The decision to re-examine HPT at two weeks of age was also at the doctor’s discretion, with 118 (43.0%) neonates re-examined, of whom 113 received additional vitamin K. At one month of age, all neonates exceeded the reference value by >40%, with no difference between the two groups, regardless of the feeding method (p=0.846). No neonates with normal HPT levels before discharge from the obstetrics department had a low HPT level at one month. None of the infants in our study presented hemorrhagic symptoms or underlying conditions that might have caused secondary vitamin K deficiency.

## Discussion

The triple-dose vitamin K prophylactic regimen was effective, and all healthy neonates achieved normal HPT levels (>40%) at one-month after birth, with no incidence of VKDB. Even if the HPT value was low one week after birth, the administration of vitamin K resulted in 91% of the neonates achieving sufficiently high HPT values at two weeks (HPT>30%), with all neonates having HPT values >40% at one month. The feeding method (breastfeeding or formula milk/mixed) did not affect HPT values at two weeks and one month after birth. In addition, there were no differences in HPT values at one month between infants who received vitamin K supplementation at two weeks and those who did not. Moreover, there was no effect of the feeding method on HPT values in the two groups, indicating that either breast or formula milk can provide adequate vitamin K. Therefore if vitamin K is added at one week of age, there is no need for additional vitamin K at two weeks. Notably, the HPT value was not measured after the one-month follow-up period. We expect the values after the one-month follow-up to remain the same, provided adequate nutrition is maintained.

According to the 2011 revised guidelines for vitamin K deficiency bleeding in infants and newborns (Modified version), the prophylactic regimen of triple doses of vitamin K is the mainstay in Japan [7]. Before the guidelines were issued, the HPT mass screening system was designed by the Shizuoka Prefectural Policy Committee for the prevention of VKDB [9], which comprised three associations in the Shizuoka Prefecture: the Department of Hygiene, the Association of Pediatrics, and the Association of Obstetrics and Gynecology. Most maternity institutions participated in this clinical trial in 1982 [15]. Throughout the trial, the triple-dose regimen became the mainstream method in the Shizuoka Prefecture and was established in conjunction with HPT screening. In 2022, the Japanese Pediatric Society recommended a three-month regimen in which vitamin K is administered once a week from one week to three months of age (13 doses of oral vitamin K2 syrup) [16]. However, the choice of method was left to the discretion of each institution. A follow-up survey after the publication of the 2011 modified guidelines [7] indicated that 62-82% of institutions continued with the triple-dose regimen, with other institutions (9-21%) adopting the weekly supplementation three-month regimen [17, 18]. Although the recent trend of each institution in Japan has not been surveyed, it is expected that the number of institutions adopting the three-month regimen has increased since the recommendation issued by the Japanese Pediatric Society in 2022 [16].

In Germany, where a triple dose of 2 mg of oral vitamin K is used, similar to Japan, the incidence rate of infantile VKDB has been reported as 0.44 cases per 100,000 live births [19, 20]. This estimate included neonates with secondary causes of VKDB, including biliary atresia, congenital α1-antitrypsin deficiency, etc. The prophylactic strategy for VKDB varies from country to country [5,21]. Vitamin K prophylaxis via intramuscular injection is considered more reliable and effective than oral administration [22-24] but is painful, and the risk of complications from injections and parental acceptance is problematic. It is also associated with an increased risk of childhood cancer [25]. Oral administration is painless and straightforward; however, the procedure is entrusted to parents, and there is concern about administration failure. The triple-dose regimen used in Japan is often administered at medical institutions, and no omissions were observed in our report. This issue is a concern in Japan due to the transition from a triple-dose regimen to a three-month one.

Hemorrhagic disease caused by VKDB was investigated in Japanese pediatric centers among infants born between 2015 to 2017. Thirteen cases of intracranial hemorrhage were reported (nutritional method: breastfeeding in 10 cases, formula milk in one case, and unknown in two cases) [8]. Of these, 11 had underlying hepatobiliary diseases such as biliary atresia, nine received a triple-dose regimen of vitamin K, and one had no underlying disease. No infants who received the three-month regimen developed intracranial hemorrhage [8]. A report from Germany, where the same regimen as Japan is adopted, showed that VKDB occurred in the triple-dose oral vitamin K regimen, while no VKDB cases were found in the three-month regimen, suggesting that the triple-dose regimen may be insufficient to prevent VKDB in infants with an underlying disease [5,19].

No significant differences in HPT values were observed between the nutritional methods (breastfeeding or formula milk/mixed) at two weeks or one month after birth. Moreover, no significant association was observed between the nutritional method and HPT values. Breast milk is the main diet of neonates; vitamin K levels in breast milk are significantly lower than those in formula milk [26,27], and the vitamin K content in breast milk varies among individuals [5,28]. The results of this study indicate that in newborns without underlying diseases, oral administration of vitamin K after birth and during the first week of life can prevent vitamin K deficiency, regardless of the nutritional method, until the first month of life.

Both HPT and PIVKA-II tests are effective in screening for vitamin K deficiency. However, PIVKA-II tests necessitate some blood volume, whereas the HPT is practical, as it can be easily measured from a small amount of blood (about 50 µL) obtained by heel prick. The standard value of HPT in adults ranges from 70% to 130% and varies with age. However, standard reference values for neonates do not currently exist. Based on previous studies, we set our therapeutic cutoff points at an HPT value of <29% at two weeks and <37% at one month. Nishiguchi advocated a threshold of 40%, extending the therapeutic window for vitamin K supplementation to enhance protection against VKDB [15]. Our study did not address the 'most effective' therapeutic criteria for vitamin K supplementation. However, our findings confirm that the triple-dose regimen is adequate to maintain the HPT value above the therapeutic threshold and prevent primary VKDB in healthy neonates regardless of the feeding method. Based on our findings, it might be possible to omit HPT screening in healthy neonates. 

The limitations of our study regarding the interpretation of the results need to be acknowledged. The long-term efficacy of the triple-dose vitamin K regimen is uncertain because the follow-up period was only one month. The regimen’s impact on secondary VKBD should also be evaluated over an extended period. Since the feeding method is reported by the parents, there is some concern about the accuracy of classifying the two groups. In addition, breast milk may vary between individuals, and the vitamin K content in formula milk may differ across products. More cases of ethnic and geographical diversity are needed to evaluate the relationship between this regimen and VKDB accurately. Although we emphasized HPT as an endpoint, we could not evaluate the risk of VKDB. In Japan, insurance reimbursements for HPT are no longer available, as companies have ceased accepting orders for HPT since 2019. We intend to continue this study following the transition to the three-month regimen and compare it with the triple-dose regimen. However, the study was terminated due to the unavailability of HPT testing.

## Conclusions

The purpose of this study was to evaluate the effectiveness of a triple-dose vitamin K regimen by retrospectively analyzing the outcomes of HPT-based vitamin K screening and to assess the utility of HPT as a screening method. The triple-dose vitamin K regimen may be sufficient in healthy neonates to attain an HPT value of >40%, regardless of feeding methods (breast milk or formula milk/mixed feeding). The triple-dose vitamin K regimen might be sufficient to lower the risk (and possibly prevent) VKBD in healthy neonates, and therefore, it might be possible to avoid HPT screening. However, since at-risk newborns, such as those with biliary atresia, are included in real-world clinical practice, this study does not support the triple-dose vitamin K regimen as a prophylactic measure. Further research is needed to establish the most appropriate method of preventing VKBD.
